# Infection Sources of a Common Non-tuberculous Mycobacterial Pathogen, *Mycobacterium avium* Complex

**DOI:** 10.3389/fmed.2017.00027

**Published:** 2017-03-07

**Authors:** Yukiko Nishiuchi, Tomotada Iwamoto, Fumito Maruyama

**Affiliations:** ^1^Toneyama Institute for Tuberculosis Research, Osaka City University Medical School, Toyonaka, Japan; ^2^Department of Infectious Diseases, Kobe Institute of Health, Kobe, Japan; ^3^Section of Microbiology, Graduate School of Medicine and Faculty of Medicine, Kyoto University, Kyoto, Japan

**Keywords:** biofilm, epidemiology, infection source, genotyping, *Mycobacterium avium* complex, non-tuberculous mycobacteria, showerhead, transmission route

## Abstract

Numerous studies have revealed a continuous increase in the worldwide incidence and prevalence of non-tuberculous mycobacteria (NTM) diseases, especially pulmonary *Mycobacterium avium* complex (MAC) diseases. Although it is not clear why NTM diseases have been increasing, one possibility is an increase of mycobacterial infection sources in the environment. Thus, in this review, we focused on the infection sources of pathogenic NTM, especially MAC. The environmental niches for MAC include water, soil, and dust. The formation of aerosols containing NTM arising from shower water, soil, and pool water implies that these niches can be infection sources. Furthermore, genotyping has shown that clinical isolates are identical to environmental ones from household tap water, bathrooms, potting soil, and garden soil. Therefore, to prevent and treat MAC diseases, it is essential to identify the infection sources for these organisms, because patients with these diseases often suffer from reinfections and recurrent infections with them. In the environmental sources, MAC and other NTM organisms can form biofilms, survive within amoebae, and exist in a free-living state. Mycobacterial communities are also likely to occur in these infection sources in households. Water distribution systems are a transmission route from natural water reservoirs to household tap water. Other infection sources include areas with frequent human contact, such as soil and bathrooms, indicating that individuals may carry NTM organisms that concomitantly attach to their household belongings. To explore the mechanisms associated with the global spread of infection and MAC transmission routes, an epidemiological population-wide genotyping survey would be very useful. A good example of the power of genotyping comes from *M. avium* subsp. *hominissuis*, where close genetic relatedness was found between isolates of it from European patients and pigs in Japan and Europe, implying global transmission of this bacterium. It is anticipated that whole genome sequencing technologies will improve NTM surveys so that the mechanisms for the global spread of MAC disease will become clearer in the near future. Better understanding of the niches exploited by MAC and its ecology is essential for preventing MAC infections and developing new methods for its effective treatment and elimination.

## Introduction

Diseases caused by non-tuberculous mycobacteria (NTM) have global importance in the public health arena. Steep increases in the worldwide incidence and prevalence of these diseases are linked with the increasing numbers of patients with pulmonary *Mycobacterium avium* complex (MAC) disease in many countries. Currently, NTM consist of more than 150 species (1), and they are globally ubiquitous in both natural and man-made environments. Pathogenic NTM can cause infectious diseases in humans, livestock, and wildlife. It is believed that NTM are generally acquired from the environment *via* ingestion, inhalation, and dermal contact, which results in lymphadenitis, pulmonary and disseminated infections, and skin and soft tissue infections. Although it is not clear why NTM diseases have been increasing, there are several contributing factors, such as, (i) an increase of mycobacterial infection sources in the environment, (ii) an increase in the number of susceptible individuals, (iii) improvements of laboratory detection techniques, and (iv) increased awareness of NTM diseases (2). These factors may work synergistically to increase the frequency of performing mycobacterial cultivations and diagnosing mycobacterial isolates to the species level. The basic principle of preventing infectious diseases is to clear pathogens from infection sources, to treat patients effectively, and to vaccinate susceptible people. However, effective methods for eradicating NTM from infection sources and hosts have not yet been established, and vaccines have not yet been developed. Notably, NTM are tolerant of chlorine-based disinfectants (3), and MAC is one of the most tolerant (4). Once a person is infected with MAC, it is difficult to eradicate the bacilli, as it requires prolonged therapy (at least 12 months of negative sputum cultures while receiving a combination of medicines, including macrolides) (5). Even after successfully completing therapy, microbiological recurrence is common (32–48% of cases), most often because of MAC reinfection (6, 7).

Four distinct subspecies are recognized in *M. avium*: *M. avium* subsp. *hominissuis* (MAH), *M. avium* subsp. *paratuberculosis* (MAP), *M. avium* subsp. *avium* (MAA), and *M. avium* subsp. *silvaticum* (MAS). In these *M. avium* subspecies, MAH is considered the clinically most important one for humans, and it often causes a chronic pulmonary disease. It is also known to be a causative agent of lymphadenitis in children and pigs. Other *M. avium* subspecies are also well-known pathogens; MAP causes Johne’s disease, a chronic granulomatous enteritis that principally affects ruminants, and MAA and MAS have mostly been isolated from birds with tuberculosis (TB)-like disease.

In this review, we focus on the infection sources of pathogenic NTM, especially MAC, in the environment. Verification of infection sources requires the identification of an identical genotype between clinical and environmental isolates. In addition, it also requires proof of the transmission routes of pathogens from the environment to the patients. However, it is difficult to identify the transmission routes and infection sources for MAC, because the diseases caused by it have long incubation periods and insidious onsets. Thus, these properties make it difficult to clarify the time of infection (5) and identify the transmission routes. Therefore, improving our understanding of the environmental ecology of MAC, particularly the niches it inhabits, is important for estimating its transmission routes and infection sources. Furthermore, population-wide genetic studies using new technologies, such as variable numbers of tandem repeats (VNTR) and next-generation sequencing (NGS), have provided new insights into the sources and routes of transmission of NTM, including MAC. For this review, we reviewed reports of community-acquired infection sources, except for case reports and nosocomial cases.

## Recent Trends in the Epidemiology of NTM Diseases

It is difficult to compare the incidence and prevalence of NTM diseases across geographic areas. Because reporting NTM disease to public health authorities is not required in most countries, studies of the incidence and prevalence of NTM disease are performed differently in different countries. To compare reports regarding changes in the incidence and prevalence of NTM disease over time in a limited geographic area, one must compare reports that used the same methods. Many epidemiological reports and reviews have shown that NTM disease have been increasing since the 1950s (8–11). Here, we summarize representative reviews on NTM and introduce recent articles published after the 2015 review article by Prevots and Marras (11).

### MAC Is the Main Driver for the Rise in Pulmonary NTM Diseases

Initially, MAC was not the predominant mycobacterial pathogen. In fact, increased numbers of *Mycobacterium kansasii* infections were reported in Wales (UK), Texas (USA), Japan, and other countries between the 1950s and 1970s (8, 12). However, in the decades that followed, the incidence of *M. kansasii* disease remained static (10, 13), while the worldwide prevalence and incidence of NTM disease increased greatly (10, 11). In Japan, Namkoong et al. (14) estimated the incidence of pulmonary NTM disease at 14.7/100,000 person-years in 2014 (14), which represents a 2.6-fold increase over the last 7 years (5.7/100,000 person-years in 2007) (15). The estimated incidence of pulmonary MAC also increased from 5.2/100,000 in 2007 to 13.1/100,000 in 2014. Most isolates were MAC (88.6%), followed by *M. kansasii*, and *Mycobacterium abscessus* (14). The last estimated incidence in 2007 was obtained by same method (15). Namkoong et al. (14) obtained an estimated incidence from 551 hospital-based surveillances between January and March 2014, and they determined both the number of newly diagnosed cases of pulmonary TB (2,327 cases) and pulmonary NTM disease (2,652 cases) that met the American Thoracic Society criteria for diagnosis of NTM disease. The authors estimated the pulmonary NTM disease incidence by multiplying the pulmonary TB incidence by the ratio of newly diagnosed pulmonary NTM cases to newly diagnosed pulmonary TB cases. Hamada et al. (16) reported the prevalence of pulmonary NTM in the West Harima area and Kyoto City in Japan from 2012 to 2013. The estimated prevalence of pulmonary NTM diseases in the West Harima area (85.4/100,000 person-years) was significantly higher than that observed in Kyoto City (23.6/100,000 person-years; *p* < 0.001) (16).

Shah et al. (2) performed a population-based survey in Wales and Northern Ireland (UK). All culture-positive NTM isolates between 2007 and 2012 were reported to Public Health England, and the annual incidence of NTM was calculated using de-duplicated, individual-level NTM data and mid-year population estimates from the Office of National Statistics (2). The annual incidence increased from 5.6/100,000 in 2007 to 7.6/100,000 in 2012. When focusing on pulmonary disease, the incidence increased from 4.0/100,000 in 2007 to 6.1/100,000 in 2012. The most frequently cultured organisms from individuals with pulmonary isolates were MAC. The incidence of pulmonary MAC increased from 1.3/100,000 in 2007 to 2.2/100,000 in 2012. Therefore, MAC is the main driver of the steep increase in the incidence of pulmonary NTM disease (2).

A high incidence (13.33/100,000 person-years, with Poisson 95% confidence intervals), of NTM pulmonary disease among adults ≥35 years of age was reported in Ontario, Canada, during 2001–2013 (17). In addition, Marras et al. (17) revealed that chronic obstructive pulmonary disease and asthma were associated with approximately ninefold and fivefold higher adjusted incidences of NTM pulmonary disease, respectively (17). A recent survey in Germany revealed an increase in the prevalence of pulmonary NTM disease from 2.3 to 3.3 cases/100,000 population from 2009 to 2014 (18).

While the incidences of NTM diseases have tended to increase, geographical heterogeneity has also been observed. In North Carolina, USA, the annual prevalence of NTM isolation did not differ significantly among the five study years (19). The authors reviewed laboratory reports of NTM isolation from North Carolina residents in three counties during 2006–2010. Among 1,033 patients, the overall NTM isolation prevalence was 15.9/100,000 persons. The prevalence of pulmonary NTM was 11.5/100,000. Most isolates were MAC, followed by *M. abscessus* complex. These reports clearly indicate that MAC is the main driver for the rise in pulmonary NTM diseases.

### A Substantial Number of Pulmonary NTM Disease Patients Have Been Identified among Patients with Suspected Pulmonary TB and Chronic TB

**Figure 1:**
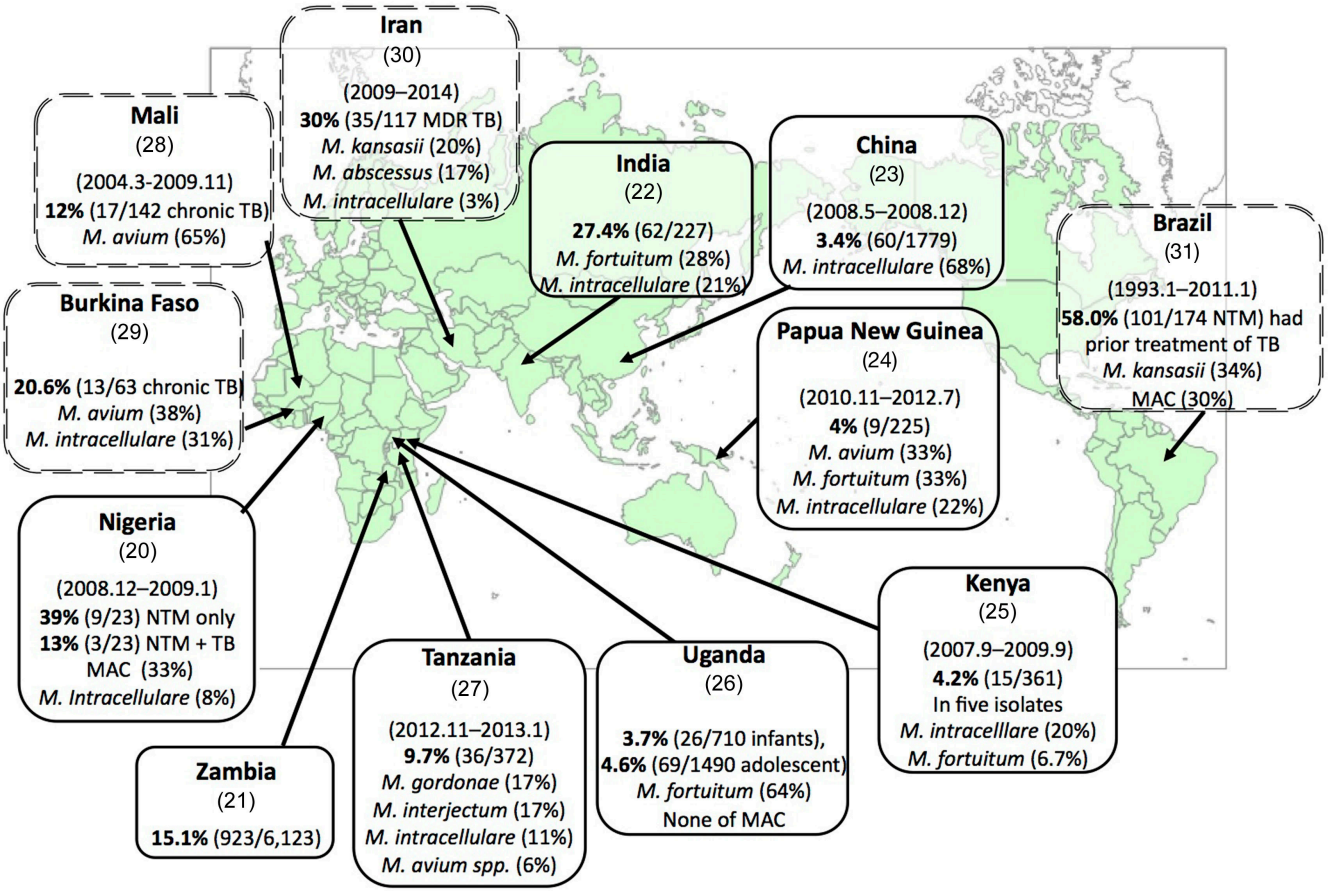
**Substantial numbers of non-tuberculous mycobacteria (NTM) disease patients have been found among suspected tuberculosis (TB) and chronic TB patients**. Each box represents the country (reference), surveillance period, percentage of emergence rate of NTM disease among suspected TB patients (NTM disease patients/suspected TB patients), and the most predominant NTM and *Mycobacterium avium* complex (MAC) species (rate of isolates). The double-dot border box represents the emergence rate of NTM disease cases among chronic TB cases or suspected multidrug-resistant-TB (MDR-TB) cases. Each reference number is shown in parenthesis below the country name.

In many countries, especially in high-burden areas for TB, the diagnosis of TB is mainly based on the detection of acid-fast bacilli in a sputum smear, as well as on their symptoms and the results of a chest X-ray. This diagnostic procedure cannot distinguish NTM from TB. In these countries, there are a substantial number of pulmonary NTM patients among patients suspected of having pulmonary TB (3.4–39%) [Figure 1, single-border box (20–27)]. Diagnosed suspected TB patients usually receive an anti-TB treatment. However, NTM disease patients are not cured by a 6-month anti-TB treatment, and they are then considered to have chronic TB or multidrug-resistant-TB (MDR-TB). Among these chronic TB and MDR-TB patients, 12–30% of them were found to suffer from NTM [Figure 1, double-dot border box (28–31)]. These facts highlight several problems: (i) NTM disease patients who were diagnosed with TB could not receive appropriate treatment for NTM disease; (ii) annual TB reports contain non-negligible errors; and (iii) this has resulted in unnecessary expenses for TB treatments. Thus, a reliable, low-cost mycobacterial diagnostic method that results in species-level identification is urgently required.

### Mycobacterial Species Responsible for Pulmonary NTM Disease in Different Geographical Areas of the World

There are differences in the relative abundances of mycobacterial species that cause NTM diseases across geographic areas (11, 32, 33). In many countries, the most frequently reported mycobacterial species is MAC. In Japan and Oregon, USA, MAC has been reported to account for 88.8% (14) [up from 73.7% in 1997 (34)] and 88% (35), respectively, of all cases of NTM diseases. In Eastern Asia, MAC accounts for 68% of all cases of NTM diseases (32). In Europe, Hoefsloot et al. (33) collected pulmonary NTM isolation and identification results from laboratories. MAC was isolated more frequently in Northern Europe (44% of all mycobacteria) than in Southern Europe (31%). As reviewed by Prevots and Marras (11), MAC was the most common species complex (64–85% of cases) in North America, followed in most studies by *M. abscessus/chelonae* (3–13%), *M. xenopi* (1–23%), *M. fortuitum* (<1–8%), and *M. kansasii* (<1–6%). In Central and South America, MAC was generally most common, and *M. kansasii* was also reported frequently (11).

However, MAC was not predominant in some areas. In French Polynesia, which is located in the South Pacific and consists of 118 islands, 67 of which are inhabited (population = 274,000) (36), the most frequently isolated species was *M. fortuitum* complex (42/87, 48.3%), followed by *M. abscessus* complex (32.2%), *M. mucogenicum* complex (9.2%), and five MAC isolates (5.7%). In Larissa, Greece, *M. fortuitum* was also predominant (30.8%), followed by *M. gordonae* (22.7%) and *M. peregrinum* (12.0%) during 2003–2013. *M. avium* (2.1%) and *Mycobacterium intracellulare* (1.8%) were reported infrequently (37). In India, *M. fortuitum* (40%, 6/15 patients) was predominant, and MAC was not detected (38). A subsequent study in India showed that *M. intracellulare* (*n* = 32, 24%) has become the most predominant species, and *M. fortuitum* (*n* = 19, 14.3%) was still dominant among 133 NTM strains that were isolated during June 2005–May 2008 (39).

## The Environmental Niches of MAC and Other NTM

Table 1 shows MAC and other NTM that were isolated from water, biofilm, soil, and dust samples. Although NTM were isolated worldwide, the isolation of MAC varied across geographic regions.

**Table 1:** **Summarized detection rates of MAC and other NTM in the environment**.

Region	Country/local (dates)	Number of households^a^ and total samples	Kinds of samples	Environmental sampling sites and number of samples	Detection method	Species identification method	Detection rate^b^ (S, samples; H, households)	Rate of MAC	Reference
East Asia	Japan/Osaka	40 (H) 166 (S)	Biofilm	Bathroom drains (38), kitchen drains (39), bathtub inlets (27), showerheads (39), showerheads, inner (23)	NTM-specific 16SrRNA qPCR	*Mycobacterium avium*-specific 16SrRNA qPCR	NTM 38.6% of S (64/166)	*M. avium* 12.5% of H (5/40)	(40)
	
	Japan/Kyoto (2007.1–2011.9)	135 (H) 135 (S)	Soil	Residential yards soils (79), potting soils (49), agricultural farm soils (7)	Culture	Multiplex PCR for MAC	MAC 48.9% of S (66/135)	*M. avium* 28.1% of S (38/135) *Mycobacterium intracellulare* 26.6% of S (36/135)	(41)
	
	Japan/Osaka (2005.1–2007.7)	29 (H) 162 (S)	Water Biofilm	Shower waters (29), bathing waters (26), showerheads (29), showerheads, inside (24), bathtub inlets (25), bathroom drains (29)	Culture	MAC-specific PCR	MAC 20.4% of S (33/162) 51.7% of H (15/29)	*M. avium* 19.8% of S (33/162) 51.7% of H (15/29) *M. intracellulare* 0.6% of S (1/162) 3.4% of H (1/29)	(42)
	
	Japan/Osaka (2004.1–2004.12)	92 (H) 704 (S)	Water Biofilm Dust	Shower waters (89), kitchen tap waters (91), bathing waters (86), shower heads (76), bathroom drains (92), kitchen sink drains (92), washbasin drains (92), dusts from air conditioners (86)	Culture	16S–23S ITS sequence	MAC 1.6% of S (11/704) 10.9% of H (10/92)	*M. avium* 7.6% of H (7/92) *M intracellulare* 3.3% of H (3/92)	(43)

Oceania	Australia	20 (H)	Water Biofilm	Water samples from kitchen, bathroom, and shower taps, rainwater tanks, and swimming pools Swabs were taken from inside all taps and showerheads	Culture	Multiplex PCR	Pathogenic NTM 95.0% of H (19/20)	*M. avium* 5% of H (1/20)	(44)

North and Central America	USA	41 (S)	Water	6 sampling sites with different distance from a point of entry to the distribution system	qPCR		NTM 88% of site (36/41)		(45)

	USA	51 (S)	Biofilm	Kitchen faucet biofilm (51)		MAC MAP Specific qPCR^c^	MAP 11.8% of S (6/51)	*M avium* 35.3% of S (18/51 *M intracellulare* *56.9% of S (29/51)*	(46)
	
	USA/Crow reservation, Mon	57 sites	Water Biofilm	Kitchens or restrooms tap water, biofilm inside of the faucets They were supplied by municipal systems (16) and by ground water wells (41)	Culture and 16S rRNA gene PCR	16S rRNA gene sequencing (>95% similarity)	NTM 35.1% of sites (20/57)	*M. avium* 1.8% (1/57) by culture 12.3% (7/57) by PCR	(47)
	
	USA	73 (H) 782 (S)	Water Biofilm	Water and biofilm samples from 80 households	Culture	ITS and 16S rRNA gene sequencing	MAC and related species 24.2% of S (182/752) 56.1% of H (41/73)	*M. avium* 19.1% of S (144/752) *M intracellulare* None	(48)
	
	USA/New York (2001–2011)	8 (H) 88 (S)	Water Biofilm	Hot and cold water (43), biofilms from water taps and showerheads (31), filters (6)	Culture	ITS or 16S rRNA gene sequencing	NTM 39.8% of S (35/88)	*M. avium* 37.5% of H (3/8)	(49)
	
	USA (2007–2009)	37 (H) 394 (S)	Water Biofilm Filter Soil	Water (47) Biofilm (46) Filters (4) Soil (17)	Culture	Nested PCR for 16S rRNA, PCR-RED of *hsp65*^d^	NTM 27.7% of S (109/394) 59.5% of H (22/37)	*M. avium* (10) *M. intracellulare* (10)	(50)
	
	USA/nine cities	45 public buildings >6,090 clones	Water Biofilm	Showerheads (45) Water Biofilm Repeatedly sampled (2–3 times at 4 sites)	16S rRNA gene-based clone libraries		NTM 28.1% of total 30.4% of clones from biofilm (1,051/3,454) 11.4% of clones from water (131/1,146)	*M. avium* (351 clones) *M. intracellulare* (5 clones)	(51)
	
	USA, Canada	21 (H) 81 (S)	Soil	Personal potting soil (79) Commercial potting soil (2)	Culture	16SrRNA gene sequencing	NTM 34.6% of S (28/81) including commercial potting soil	*M. avium* 15.2% of S (12/79) *M. intracellulare* 21.5% of S (17/79)	(52)
	
	USA/San Francisco (1990.10–1992.8)	290 (H) 1,082 (S)	Water Soil Food	Tap water (385) Bottled water (77) Water from outside of household Soil (157) Food (397)	Culture	Probe method	NTM 22.4% (242/1,082) of S 17% of water (90/528) 3 of foods (12/397) 89% of soil (140/157)	*M. avium* (45) 4.2% (45/1,082) of S 0.19% of water (1/528) 0.25% of foods (1/397) 27% of soil (43/157)	(53)
	
	Mexico/Mexico City (2008.11–2009.10)	5 (H) 120 (S)	Water	Kitchen tap water, every month	Culture	PCR-RED of *hsp65* 16S rRNA gene, *rpoB* sequencing	NTM 15.8% (19/120) of S	*M. avium* 2.5% (3/120)	(54)

Europe	Greece/Larissa (2010–2013)	30 localities 3,360 (S)	Water	Drinking water samples	Culture	Genotype CM kit	NTM 11.2% of S (376/3,360)		(37)
	
	Germany/Berlin and other federal stations	130 (S) 66 indoor 64 outdoor	Water Biofilm Soil Dust	Water (40); tap water, lake, river, fountains, rain puddles Biofilm (19); sanitation facilities, well wallings, filter units, river and lake borders Soil (41); flower pots, gardens, play grounds, urban spaces, forests Dust (30); vacuum cleaners, room surfaces, air filters	Culture	*M. avium* specific, subspecies specific PCR	MAH 13.8% of S (18/130) 20% of soil (8/41) 33% of dust (10/30)	Same as on the left	(55)
	
	The Czech Republic	38 (H) and others 124 (S)	Biofilm	Household drinking water tank sediments (38) from four drinking water supply systems, dam sediments (52), water treatment plant sludge samples (34)	Culture qPCR	MAC-specific PCR 16S rRNA gene sequencing	NTM 33.9% of S (42/124) by culture 76.7% of S (92/124) by PCR	MAH 0% by culture 24.2% by qPCR	(56)
	
	The Czech Republic	2 (H) 55 (S)	Water Soil Dust Others	Soils (44), cobwebs (4), dusts (2), water (1), compost materials (2), a moss (1), leaf (1)	qPCR Culture	Triplex qPCR 16S rRNA gene sequencing	MAH 42.8% of S from patient 1 47.6% of S from patient 2	Same as on the left	(57)
	
	Italy/Latium region and Calabria region	20 (H) and others 42 (S)	Water	Tap waters of hospitals (22) and households (20)	Culture	PCR-RED of *hsp65*	NTM 61.9% of S (14/42)	*M. intracellulare* (frequently isolated)	(58)
	
	Greece/Trikala City (2007.1)	2 (H) and others 22 (S) 112 (clones)	Water	Tap water (2) Drilling wells (13) Water treatment tank (7)	16S rRNA gene-based clone libraries		NTM 5.3% of clones (6/112) 26.1% of clones from tap water (6/23)		(59)

Africa	Uganda (2008.9–2009.1)	231 (H) 310 (S)	Water Soil Animal feces	Household drinking water (130) Water from valley dam and stream (56) Soil from animal kraal (44) Soil from water source (47) Animal feces (33)	Culture	INNO-Lipa test 16S rRNA gene sequencing	NTM 15.5% of S (48/310)	MAC (5) *M. intracellulare* (9)	(60)

Middle East	Iran (2015.6–9)	110 (S)	Water	Tap water, source water	Culture	16S rRNA gene and *rpoB* sequencing	NTM 32% of S (35/110)	MAA (1) *M. intracellulare* (1)	(61)
	
	Iran/four suburbs	4014 (S)	Water Soil	Tap water (260) Damp water (290) Running water on raceway (1,396) Soil (2,068)	Culture	PCR-RED of hsp65 and ITS gene	NTM 21.4% of S (862/4,014)	*M. avium* 4.7% of NTM (20/862)	(62)

*^a^Number of households that provided samples (H) and a total number of samples (S)*.

*^b^Detection rates are represented per sample (S) and per household (H)*.

*^c^qPCR assays targeting partial 16S rRNA gene sequences for M. avium and M. intracellulare and targeting IS900 and IS1251 for M. avium subsp. paratuberculosis*.

*^d^PCR-RED analysis: PCR amplification and analysis of restriction endonuclease digestion fragments (PCR-RED) of the heat-shock protein 65 (*hsp65*) gene*.

*ITS, internal transcribed spacer; MAC, Mycobacterium avium complex; NTM, non-tuberculous mycobacteria; MAP, M. avium subsp. paratuberculosis; MAH, M. avium subsp. hominissuis*.

### Frequent Recovery of MAC from Tap Water and Bathrooms in North American and Japanese Households, Respectively

Numerous studies have shown that showerheads and tap water, the end-points of drinking water distribution systems (WDSs), are MAC reservoirs in households (Table 1). Pathogenic NTM, including *M. avium*, were obtained from the interior surfaces of 45 showerheads from 9 cities in the USA using a culture-independent method (51). The results showed the persistence of particular sequence types, e.g., *Mycobacterium* spp. (28.1% of total), while *M. avium* accounted for 30% of the mycobacterial biofilm samples. In addition, NTM and other opportunistic human pathogens were enriched to high levels in many showerhead biofilms. Wallace et al. (48) evaluated MAC household water isolates from 3 published studies and 37 additional MAC respiratory disease patients. The water and biofilm samples were obtained from 752 individual sites in 80 households of 73 NTM patients. Species identification was initially performed using non-sequencing methods with confirmation by internal transcribed spacer (ITS) and/or 16S rRNA gene sequencing. Although *M. intracellulare* was identified by non-sequencing methods in 41 household water/biofilm samples, ITS sequencing revealed that none of the samples contained *M. intracellulare* and that 30 samples were *M. chimaera*, while 8 were other MAC X species. In comparison, *M. avium* was recovered from 144 (19.1%) water/biofilm samples. These results indicate that *M. intracellulare* lung disease in the USA is acquired from environmental sources other than household water. Non-sequencing methods for NTM identification might fail to distinguish closely related species (such as *M. intracellulare* and *M. chimaera*) (48).

In rural areas in the USA, over 15 million households rely on private ground water wells for their primary drinking water sources. Richards et al. (47) examined tap water and their associated biofilm samples from a total of 57 sites from untreated groundwater (41 sites) and treated municipal drinking systems (16 sites) on the Crow Reservation in rural Montana, USA. *Mycobacterium* species were detected in samples from 20 (35.1%) of the 57 locations, in both treated municipal water (8 sites) and untreated well water (12 sites) (47). These studies showed that MAC was recovered frequently from household water in both urban and rural areas in North America.

In Japan, MAC has been isolated only from bathrooms (11 isolates), while it has not been isolated from kitchen tap water, wash basins, and other sites in households of 49 pulmonary MAC disease outpatients and 43 healthy volunteers (43). The incidence of MAC in the bathrooms of patients was significantly higher than that in healthy volunteers’ bathrooms (*p* = 0.01). Of the 11 MAC isolates, 2 were isolated from showerheads, 3 from shower water, 4 from bathtub water, and 2 from drain outlets (43). An additional survey of 29 pulmonary MAC disease patients revealed the polyclonal colonization of MAC in their residential bathrooms (42). Particularly, it was found that MAC predominantly colonized bathtub inlets. A bathtub inlet is a special piece of equipment in Japanese-style bathtubs, and it is located inside the bathtub. Hot water is supplied from the bathtub inlet, which is connected to a bath boiler or a hot-water supply. Another study in Japan also detected *M. avium* from bathtub inlets in 5 residences using a culture-independent method (40) after collecting specimens from 5 sites in 40 healthy volunteers’ homes (Table 1).

### Where Do MAC and Other NTM Organisms in Tap Water Come From?

Household tap water is provided by treatment plants *via* drinking WDSs. Do NTM organisms also travel from untreated water through drinking water treatment plants to household tap water? To answer this question, Klanicova et al. (56) obtained 124 samples from 4 drinking water supply systems in the Czech Republic, and they detected MAC by culture and quantitative real-time PCR (qPCR) methods (56). The samples included 52 dam sediments, 34 water treatment plant sludge samples, and 38 tap water household sediments. They showed that 92 (74.2%) of the samples analyzed were *M. avium* subspecies-positive according to the qPCR results, and the subspecies detected included MAP (36.3%), MAA (13.7%), and MAH (24.2%). The frequency of the *M. avium* subspecies-positive samples indicated a statistically significant declining trend along the route leading from the dam to the water treatment station to the households (the *p*-value of theχ^2^ test for the trend was <0.01) (56).

Kormas et al. (59) surveyed 13 water pumping wells, the water in a treatment tank, and the tap water from 2 households in Trikala City, central Greece, in 2007 using a culture-independent method. They obtained a total of 191 clones. While *Actinobacteria*, which are closely related to NTM, did not appear in the clonal libraries resulting from the pumping wells and the treatment tank water, they were dominant in the tap water. Six clones were closely related to NTM, including *M. gordonae, M. mucogenicum, M. gadium, M. neglectum, M. sherrisii*, and an unidentified *M*. sp (59). Another group surveyed 41 water samples from 6 sampling sites located at different distances from a point of entry to a WDS in the USA (45). They detected *Mycobacterium* spp. in 88% of the samples. The densities of *Mycobacterium* spp. were generally higher (324-fold) for distal sites relative to the entry point of the distribution system.

These studies suggest that mycobacterial communities are likely to accumulate at the end of a WDS. The NTM might travel from raw water through the WDS to household tap water. Although the presence of NTM in household tap water may be partially responsible for the dissemination of these organisms, the recent global increase in pulmonary NTM disease patients remains unexplained. Furthermore, MAC was recovered from bathrooms, but not from kitchen tap water, in Japan (40). This suggests the existence of a transmission route other than the WDSs.

### Frequent Recovery of MAC from Soil in Europe

Soil and house dust are also reservoirs for NTM. In Europe, MAC and other NTM are isolated frequently from soil, compared with water and biofilm samples (Table 1). In Larissa, Greece, Dovriki et al. (37) analyzed 3,360 drinking water samples from 30 localities of 367 NTM patients’ residence areas during 2010–2013 using a culture method. Interestingly, NTM were not found in water samples where the concentration of residual chlorine was greater than 0.5 mg/L. When the residual chlorine concentrations ranged from 0.0 to 0.5 mg/L, NTM were found in 11.2% (*n* = 376) of the samples, and *M. gordonae, M. fortuitum*, and *M. peregrinum* accounted for 41.0, 38.3, and 6.9%, respectively, of these isolates (37). In Germany, Lahiri et al. (55) collected 130 samples from indoor (66 samples), outdoor (25 samples), and countryside samples (39 samples) containing water, biofilm, and soil samples. The NTM isolates were then recovered by a culture method. Interestingly, MAH was predominant in the soil and dust samples, while MAH was not identified in the water and biofilm samples. Furthermore, MAH was isolated from 24% of the indoor samples, while only 3% of the outdoor samples yielded MAH isolates. A similar result was obtained when samples were collected from areas with frequent human contact (home dust, soil from flower pots, gardens, and playgrounds, tap water, and biofilms from sanitation facilities, filter units, and aquariums) and compared with samples involving less probable human contact (55). In the Czech Republic, Kaevska et al. (57) determined the presence of MAH and MAA, as well as other NTM, in environmental samples including water, soil, soil fertilized with chicken droppings, dust, cobwebs, compost materials, and moss and leaves from two residences of children who were diagnosed with MAH cervical lymphadenitis. A triplex qPCR examination revealed the presence of MAH/MAA in the potting soil, garden soil, and dust from both the residences (57).

High recovery of MAC from water samples in Europe has also been reported. In Italy, Briancesco et al. (58) collected 42 water samples from taps of 22 hospitals and 20 households. The rates of NTM contamination were 60% for household tap water and 73% for hospital water samples. The most frequently isolated strains were *M. intracellulare, M. genavense*, and *M. haemophilum* (58).

Isolates of NTM have been recovered from soil samples not only in Europe, but worldwide. In USA, De Groote et al. (52) analyzed 79 soil samples from 26 pulmonary NTM disease patients’ households. They demonstrated the aerosolization of contaminated NTM in soil using the following method. The soil samples were dropped off, aerosolized particulates were inoculated, and NTM were isolated. The most frequent pathogens in patients, such as *M. avium* and *M. intracellulare*, were also the most abundant mycobacteria in the soil (52).

In Japan, MAC and other NTM were detected in soil and house-dust samples. Ichiyama et al. (63) examined 33 samples from soil (5 samples), ditch-mud (4 samples), house dust (22 samples), and river water (2 samples), and they recovered NTM from 5 soil samples, 4 ditch-mud samples, 17 house-dust samples, and 1 river-water sample. In total, 247 MAC isolates and 78 *Mycobacterium scrofulaceum* isolates were recovered (63). A recent study showed that MAC strains were recovered from 48.9% of residential soil samples in households of 100 pulmonary MAC patients and 35 non-infected control patients. The frequency of MAC recovery did not differ among soil types or among patients, regardless of the presence of pulmonary MAC disease or the identity of the infecting MAC species (41). In addition, a subsequent report showed that high exposure to soil (≥2 h/week) was associated with polyclonal and mixed mycobacterial MAC infections in pulmonary MAC disease patients (64).

In Uganda, Kankya et al. (60) investigated 310 samples (soil, water, and fecal samples from cattle and pigs) from pastoral communities, and they detected NTM in 48 samples (25.3% of the soil samples, 11.8% of the water samples, and 9.1% of the fecal samples). Of these samples, *M. fortuitum*–*M. peregrinum* complex (12 isolates), MAC (14 isolates), *M. gordonae* (5 isolates), and *M. nonchromogenicum* (5 isolates) were the most frequently detected mycobacteria. MAC was recovered from drinking and natural water, as well as soil samples, but not from fecal samples. The authors warned that many patients might be at high risk of NTM infections because of the high incidence of HIV/AIDS in Uganda (60).

**Figure 2:**
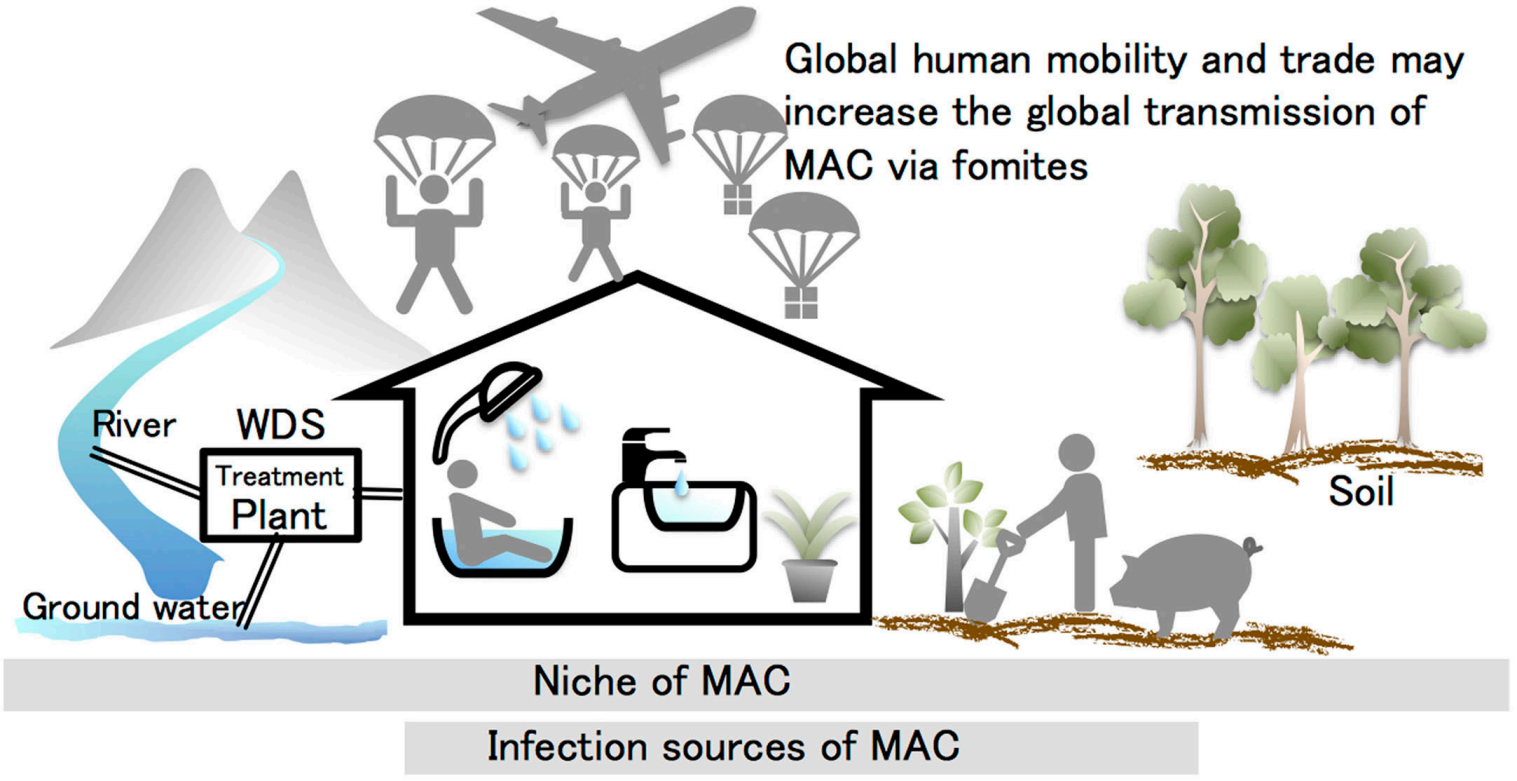
**Hypothesis for the causes of the steep global increase in pulmonary *Mycobacterium avium* complex (MAC) diseases**. MAC organisms are ubiquitous in the environment. Many studies have indicated that these organisms tend to occur in the household. Tap water, bathrooms, potting soil, and garden soil are the infection sources identified by matching the genotypic profiles of clinical and environmental isolates. The mycobacterial transmission routes are considered to occur naturally through the water distribution system (WDS) to the household. This transmission route may be partially responsible for infection cases, but it cannot explain the recent global increase in patients presenting with MAC diseases. This suggests that the transmission of MAC and other non-tuberculous mycobacteria organisms are likely to be linked with human activities. Global human mobility and trade may promote the global transmission of MAC *via* fomites.

The niches used by MAC and other NTM organisms are soil, water, and dust, and many reports indicate that MAC species tend to occur in households (Figure 2). The household niches exploited by MAC, which have been shown to vary regionally, include household tap water in North America (47, 48), bathrooms in Japan (40, 42, 43), and soil in Europe (55, 57). Despite these regional differences, the commonality among them is that MAC was frequently isolated from indoor samples and from samples collected from areas with frequent human contact (40, 42, 43, 47, 48, 51, 55, 57). This suggests that individuals may carry NTM organisms that also attach to their belongings. Therefore, acquiring information about the regional differences in NTM organisms, where they accumulate in houses, and whether other niches for them exist is now a priority for research in this area. Accumulating such data would help to elucidate the mechanisms involved in the global spread of MAC. It is feasible that new infection sources would be identifiable in such data.

## Ecology of MAC and Other NTM Organisms in Environmental Niches

Long-term colonization of showerheads and tap water indicates that MAC and other NTM species attach to surfaces, withstand water flow, and grow inside of showerheads and plumbing pipes. Indeed, a longitudinal study verified colonization by a single clone at drinking water point-of-use sites for up to 26 months (65). Lehtola et al. (66) demonstrated that *M. avium* can withstand water flows, as determined using a Propella biofilm reactor in a laboratory experiment (66). These facts have led to the common understanding that NTM form biofilms, even though mycobacteria do not possess appendages such as flagella and pili. These appendages have been reported to play an important role in the first steps of biofilm formation, such as chemotaxis and attachment to surfaces (67). This raises the following questions: how can slow-growing mycobacteria attach to surfaces and form biofilms by overcoming competition with other fast-growing microbes? In addition, can mycobacteria form multispecies biofilms that contain fast-growing microbes? If so, how can mycobacteria grow inside a multispecies biofilm with other fast-growing microbes? Although many studies of mycobacterial biofilms have been reported, these questions remain unanswered.

Another possibility for survival in the environment is within a free-living amoeba where NTM might be protected from attack by fast-growing microbes. As is well known for *Legionella pneumophila*, free-living amoebae can provide the bacteria with an ideal environment in which to multiply. In addition, free-living amoebae may also promote survival within macrophages. It has been reported that pathogenic mycobacteria can proliferate within free-living amoebae *in vitro*, and it has been show that *M. avium* replicates within amoebal vacuoles and exists at the outer walls of the double-walled cysts of *Acanthamoeba castellanii* (68), *Acanthamoeba polyphaga* (69), and *Tetrahymena pyriformis* (70).

To better understand the ecology of these microbes it is important to observe directly *Mycobacterium* biofilm formation and free-living amoebae that harbor NTM in the environment. Feazel et al. (51) used scanning electron microscopy to directly observe a biofilm that formed inside a showerhead. The resulting image showed that microbes were clumped and embedded in extracellular material. Recently, Gomez-Smith et al. (71) investigated a water main biofilm in Saint Paul, MN, USA. They directly observed the biofilm inside the water main, and they assayed the quantity and composition of bacterial biofilms using qPCR targeting the 16S rRNA gene, as well as NGS (71). They compared two types of water mains, unlined cast-iron and cement-lined cast-iron; the former possessed corrosion tubercles, while the latter did not. They revealed that the biofilm communities predominantly contained a genus of *Mycobacterium* at the main wall-bulk water interface (25–78% of the community), regardless of the water main age, estimated water age, water main material, or the presence of corrosion products. The two dominant mycobacteria were *M. frederiksbergense* and *M. aurum*. In addition, mycobacteria were detected from the surface tubercles, but not from underneath the tubercles. These results show that NTM can form biofilms *via* attachment to inanimate surfaces or to the biofilms formed by other microbes under various conditions. NTM seems to form biofilms by overcoming competition with other fast-growing microbes, but the mechanism underlying this ability remains unclear.

Recent studies showed that NTM survive and proliferate within amoebae in the environment. Thomas et al. (72) investigated the presence of free-living amoebae and amoebae-resistant bacteria at various stages of a drinking water plant that was fed with river water in France. *M. mucogenicum* was directly recovered from an *Echinamoeba*-related amoeba that was isolated from ozone-treated water. *Echinamoeba*- and *Hartmannella*-related amoebae were mainly recovered in the drinking water plant. *Acanthamoeba*- and *Naegleria*-related amoebae were recovered from the river water and sand filtration units (72).

Delafont et al. (73) reported the isolation of free-living amoebae and amoebae-associated NTM from 25 end-point water samples that were collected monthly from September 2012 to September 2013 in Paris, France. The cultivable amoebae were recovered from 174 (69.3%) of 251 water samples, and total DNA at the amoebal migration front were extracted from 129 out of 174 samples; 113 out of 129 samples (87.6%) were positive for mycobacteria. They also observed numerous acid-fast bacteria inside amoebae, especially *Acanthamoeba* and *Protacanthamoeba*, using microscopy. They screened 42 samples harboring a single amplification for amoeba and mycobacterial species identification. Their identification based on 18S rRNA and *rpoB* sequencing, respectively, revealed the presence of various free-living amoebae, such as *Vermamoeba vermiformis, Protacanthamoeba bohemica*, and *Acanthamoeba* spp. The highest number of identified mycobacterial species were related to *Mycobacterium llatzerense* (>90%), followed by *M. chelonae, M. aromaticivorans, M. phocaicum*, and *M. mucogenicum* (73).

In the environment, NTM are free-living, but they also live in biofilms and inside protozoa, and these three survival styles may affect each other. However, MAC and other pathogenic NTM species have not been directly identified inside protozoa yet. Further investigation of the ecology and interactions among NTM survival styles, the mechanism of overcoming competition with other fast-growing microbes, including the regulatory systems that govern them, is needed. These investigations are crucial for gaining better understanding of NTM infection mechanisms and for developing a strategy to eradicate these pathogens from niches and hosts.

## Infection Sources for MAC in the Environment

Respiratory infections are thought to be caused by inhalation of contaminated aerosols. It has been shown that aerosolized shower water (44) and aerosolized soil (52) contain MAC and other NTM organisms. Contaminated aerosols have also been reported to be produced by pool surfaces in a hospital therapy pool (74). Therefore, NTM niches could be important infection sources. The verification of infection sources requires the identification of the identical genotype in clinical and environmental isolates. Table 2 presents the results of studies in which the clinical and environmental isolates had identical genotypes.

**Table 2:** **Studies of infection sources in which the genotype of isolates matched between household environmental and clinical specimens**.

Region	Country (dates)	Environmental sources	No of patients	Clinical disease	Clinical samples	Genotyping	Rate of matching	Reference
East Asia	Japan (2007.1–2011.9)	Soil in pot and in yard	100	Pulmonary *Mycobacterium avium* complex (MAC) disease	Sputa	Variable numbers of tandem repeats	*M. avium* 7.5% (5/67) *M. intracellurare* 3.0% (1/33)	(41)
	
	Japan (2005.1–2007.7)	Bathrooms Bathtub inlet	29	Pulmonary MAC disease	Sputa	Pulsed-field gel electrophoresis (PFGE)	*M. avium* 24.1% (7/29)	(42)
	
	Japan (2004)	Bathrooms Bathtub water	49	Pulmonary MAC disease	Sputa	PFGE, restriction fragment length polymorphisms (RFLPs)	*M. avium* 4.1% (2/49)	(43)

Oceania	Australia	Bathrooms Tap water Aerosol from shower water	20	Pulmonary non-tuberculous mycobacteria (NTM) disease	Respiratory samples	Repetitive element palindromic PCR (Rep PCR)	NTM 20% (4/20) *Mycobacterium abscessus* (2) *Mycobacterium kansasii* (1) *M. lentiflavum* (1) 20% (4/20)	(44)

North America	USA (2001–2011)	Tap water Filter	8	Chronic NTM rhinosinusitis	Sinus cavity samples	PFGE, rep PCR	*M. avium* 37.5% (3/8)	(49)
	
	USA	Shower water Tap water	31	NTM infection		Rep PCR	NTM 22.6% (7/31)	(50)
	
	USA	Tap water	27	*M. avium* disease	Respiratory and other sites	PFGE	*M. avium* 3.7% (1/27)	(75)
	
	USA, Canada	Aerosol from potting soil	26	Pulmonary NTM disease	Respiratory samples	PFGE	*M. avium* 3.8% (1/26)	(52)

Europe	Czech Republic	Soil in yard	2	*M. avium* subsp. *hominissuis* (MAH) cervical lymphadenitis	Surgical excision-tissues	I*S1245* RFLP	MAH 50% (1/2)	(57)

In Japan, residential bathrooms of patients with pulmonary MAC disease have been reported to be a niche of MAC, as described above (42, 43). To assess the infection sources of residential bathrooms, a genotyping comparison was performed between environmental and clinical isolates using pulsed-field gel electrophoresis (PFGE). In these studies, identical strains of *M. avium* were identified [2/49 (4%) by Nishiuchi et al. (43) and 7/29 (24%) by Nishiuchi et al. (42)]. Thus, MAC organisms in bathrooms are likely to be transmitted to the users. However, it is possible that MAC organisms might migrate from patients to their bathrooms. Fujita et al. (41) examined soil samples from 100 pulmonary MAC disease patients. Six cases (6%) showed a matching profile of VNTR between the clinical and soil isolates (41). These patients were exposed frequently to soil (≥2 h per week), suggesting that residential soils are a likely source of pulmonary MAC infections.

Thomson et al. (44) examined household water and shower aerosols of patients with pulmonary NTM disease. They chose 20 patients who had resided in the same dwelling for greater than 5 years prior to the diagnosis of NTM disease. They performed a repetitive element palindromic PCR, and they obtained identical or related properties in four cases (20%). Additionally, NTM were detected in aerosols in 9 of 18 homes (44). Tichenor et al. (49) collected household plumbing samples from eight adult outpatients who suffered from NTM-infected chronic rhinosinusitis in New York, USA. In three cases (37.5%), *M. avium* strains were isolated from households and patients, and they possessed almost identical profiles according to PFGE (49).

These studies suggest that tap water, residential soil, and bathrooms in patients’ households were infection sources (Table 2). The reported patients suffered from pulmonary MAC disease, pulmonary NTM disease, chronic NTM rhinosinusitis, and MAH cervical lymphadenitis. As mentioned earlier in this review, NTM disease is characterized by a long incubation period with an insidious onset. Therefore, these infection sources were identified after the onset of clinical signs and symptoms, not at the time of infection. Thus, these sources are “estimated infection sources,” and they possess the following common properties: (i) MAC and other NTM organisms colonize these areas; (ii) in these areas, patients regularly make contact with the infection sources and may, therefore, have frequent opportunities for contact with MAC and other NTM organisms. In general, MAC patients are at risk of reinfection and recurrence of MAC and other NTM species. Also, patients with NTM disease may experience repeat infections with different clonal strains or the same clonal strain from these infection sources. Consequently, these infection sources are a risk to patients who easily become reinfected with such pathogens. Therefore, determining the infection sources is important for preventing further infection in such patients. In addition, initiating a population-wide epidemiological survey would provide further knowledge about the infection sources for these pathogens, and this is the subject of the next Section “Population-Wide Genetic Studies.”

Mycobacterial communities are likely to gather at the infection sources in households (Table 1). A possible mycobacterial transmission route involves travel from natural reservoirs to households (e.g., WDSs). While the transmission routes may be partially responsible for the increased incidence of pulmonary MAC disease, it is difficult to explain the recent global increase in the number of patients with this disease (i.e., the globalization of pulmonary MAC disease; Figure 2). The global spread of pulmonary MAC disease might be caused by human activities, as individuals carry MAC organisms that concomitantly attach to their belongings and their living environments. De Groote et al. (52) certified the existence of NTM in a commercial soil, which supports this hypothesis (52). That is, global human mobility and trade may lead to the global transmission of MAC *via* fomites. To explore this hypothesis, further investigation of the niches used by MAC and its ecology are required. Furthermore, population-wide genetic studies and genome epidemiology should provide new insights into the sources and routes of MAC transmission.

## Population-Wide Genetic Studies

Four distinct subspecies of *M. avium* have been identified, e.g., MAH, MAP, MAA, and MAS. Turenne et al. (76) performed a comprehensive phylogenetic analysis of *M. avium via* multi-locus sequencing typing of 10 genes (8,064 bp) using 56 genetically diverse strains of *M. avium* that included all subspecies. The results showed that MAH had the highest level of genomic heterogeneity within a single subspecies. They concluded that MAH represents a diverse group of organisms from which the other subspecies, MAP, MAA, and MAS, evolved independently (76). This high genetic diversity of MAH focused attention on the phylogeographical differences of this subspecies, which could reside in different infection sources in different regions. Population-wide genetic studies using many isolates that cover different regions will be needed to ascertain the phylogeography of *M. avium*.

Until very recently, genetic studies that used many isolates were technologically limited, and it was difficult to compare data from different studies. Traditional fingerprinting methods, such as restriction fragment length polymorphisms based on the IS*1311* and IS*1245* insertion sequences and PFGE, are valuable methods for typing MAH strains. However, its use is restricted to comparing only small numbers of isolates because it is technically challenging and time-consuming, and there is a lack of inter-laboratory reproducibility, which makes it difficult to compare data from different experiments and laboratories. To overcome the drawbacks of these methods, a VNTR typing analysis of MAH has been developed (77, 78). It is a simple, PCR-based genotyping method that uses the polymorphisms of minisatellites (79, 80). The method shows a high level of reproducibility, and its digitalized data make it easy to compare results from different experiments and facilities (81). The emergence of this new genotyping method, VNTR analysis, has opened the door for global epidemiological studies of MAH.

Iwamoto et al. (82) performed a VNTR analysis of a large MAH population using 258 Japanese isolates (146 human isolates, 37 bathroom isolates, and 75 pig isolates), 68 French isolates (14 human isolates and 54 pig isolates), and 22 Finnish isolates (10 human isolates and 12 pig isolates) (82). In their following paper, they also included 77 MAH isolates from Korean patients (83). Later, Leão et al. (84) incorporated these data with their own VNTR data for humans (*n* = 28) and pigs (*n* = 69) in mainland Portugal (84). Ichikawa et al. (85) also performed a VNTR analysis of MAH isolates from different regions, i.e., East Asia [Japan (*n* = 94) and Korea (*n* = 98)], Europe [Netherlands (*n* = 27) and Germany (*n* = 10)], and the USA (*n* = 32) (85). These studies revealed the following global epidemiological aspects of MAH. (i) The isolates from Japanese patients showed a high degree of genetic similarity with the Korean isolates, whereas their similarities with the European and USA isolates were quite low. (ii) The MAH isolates from Japanese patients showed a low degree of similarity with the pig isolates, whereas the isolates from European patients showed a high degree of similarity with the pig isolates. (iii) The pig isolates from Japan were closely related to the European isolates from both humans and pigs.

These results indicate that the prevalent strains vary across geographical regions. This implies the existence of different infection sources, routes of transmission, and clinical manifestations in different regions. The high genetic relatedness between human and pig isolates in European countries supports the view that there is a common source of MAH infection for pigs and humans, or that pigs are vehicles for human infections in these countries (86). However, this is not true in Japan because of the low genetic similarity between human and pig isolates. In contrast to the human cases, the pig isolates are more homogeneous at a global level. Attractive hypotheses for this global similarity of pig isolates are (i) there are common infection sources for pigs at the global level, such as piggery bedding materials like peat, sawdust, and straw and (ii) pig-derived MAH strains have been globally distributed through the importing/exporting of pigs, including breeding pigs (82).

A population-wide genetic study using the VNTR method revealed that Japanese human isolates can be divided into two major clonal complexes, one of which is highly coexistent with bathroom isolates, while the other is mainly formed by human isolates that coexist less frequently with bathroom isolates (82). The former clonal complex suggests that bathrooms are one of the major reservoirs of MAH in Japan, which exposes humans to MAH. This is consistent with the previous reports that were mentioned in the Section “Infection Sources for MAC in the Environment.” However, the existence of the other clonal complex implies that there are infection sources other than bathrooms. Fujita et al. (41) performed a VNTR analysis for 47 *M. avium* clinical isolates and 41 soil isolates. They concluded that residential soils are a likely source of pulmonary MAC infection in Japan because five pairs of clinical isolates and corresponding soil isolates showed identical VNTR patterns, and both human and soil isolates are mixed up in the phylogenetic tree constructed from their data (no distinct major clusters for clinical or soil isolates) (41). Although there are no reports that directly compared the data by Iwamoto et al. and Fujita et al., it is likely that the soil isolates belong to the clonal complex with fewer numbers of bathroom isolates. Further detailed investigation is required to clarify this issue. Iakhiaeva et al. (87) analyzed 416 MAH isolates from 121 patients and 80 household water (biofilm) samples in northeast Texas and a Philadelphia suburb as well as a small number of isolates from around the USA (87). Forty-nine VNTR types were identified among them, and 23% of the types were found in both the patient and household isolates. Most of the patients with the same VNTR types were found within the same city. Moreover, the same VNTR type was detected in local commercial water supplies and patient households. These results emphasize the need for a risk analysis of MAH in drinking water.

Further studies focusing on the global phylogeographical distribution of MAH would clarify the global commonality and local characterization of MAH infection sources and provide a clue for achieving better control of MAH infections. To facilitate such studies, the creation of an international VNTR database is undoubtedly required. The critical obstacle for the creation of a global database is a lack of standardization for VNTR locus sets. Several MAH locus sets have been reported, but no unified combination of locus sets has been used in different studies by different researchers (77, 78, 82, 87, 88). This makes the data comparison difficult among different studies. Therefore, an international collaboration to set up a global standard for VNTR locus should be established immediately.

## New Whole Genome Sequencing (WGS) Techniques Deliver a New ERA for NTM Surveys

There is an emerging new wave in the field of molecular epidemiological studies, genome epidemiology, which uses WGS technology (89–91). This method offers a detailed assessment of the single-nucleotide polymorphism (SNP)-level diversity and genetic relationships among isolates. Therefore, it can correctly classify strains as being the “same” or “different” with an extremely high level of accuracy that cannot be achieved by current genotyping methods.

The first application of WGS techniques for an NTM survey was performed by Bryant et al. (92) who defined the acquisition mechanisms of *M. abscessus* subsp. *massiliense* in individuals with cystic fibrosis (CF) (92). They analyzed 168 consecutive isolates of *M. abscessus* from 31 patients. A phylogenetic analysis revealed two clustered outbreaks of near-identical isolates of *M. abscessus* subsp. *massiliense* (from 11 patients) that differed by less than 10 bp. This variation represents less diversity than that seen within isolates from a single individual, strongly indicating between-patient transmission. Although the exact transmission route is yet to be established, their epidemiological analysis suggests that it could be indirect (92). Harris et al. (93) also applied WGS techniques to define *M. abscessus* acquisition mechanisms (93). They analyzed 27 isolates from 20 patients. A maximum likelihood phylogenetic tree showed three distinct clades corresponding to three subspecies. Twenty isolates from this study were *M. abscessus* subsp. *abscessus*, six were *M. abscessus* subsp. *massiliense*, and one was *M. abscessus* subsp. *bolletii*. Apart from these, the minimum distance between any 2 isolates from this study was 34 SNPs, indicating that there was no cross-transmission of *M. abscessus* within the hospital, except between 1 sibling pair (93).

Another large-scale survey using WGS was reported recently (94). The authors investigated whether cross-infection, rather than independent environmental acquisition, might be the major source of infection for *M. abscessus*. They performed a WGS analysis of 1,080 clinical isolates of *M. abscessus* obtained from 517 patients in UK CF clinics and their associated regional reference laboratories, e.g., CF centers in the USA, the Republic of Ireland, Europe, and Australia. They illustrated that most *M. abscessus* infections were acquired through the transmission, potentially *via* fomites and aerosols, of recently emerged, dominant circulating clones that have spread globally.

These reports confirm the ability of WGS techniques to successfully investigate infection sources for NTM. WGS has great potential to reshape our understanding of the infection sources and transmission dynamics of MAC and other NTM organisms in the near future.

## Prevention of Colonization and Disinfection of Niches

How can we prevent MAC from colonizing environmental niches? One possibility is to ensure that such niches remain dry. Nishiuchi et al. (42) performed a questionnaire survey. They asked volunteer pulmonary MAC patients, who provided water and biofilm samples, about the maintenance of their bathrooms. Their results showed that promptly draining bath water and increasing ventilation times effectively decreased MAC recovery from bathrooms. Thus, drying the bathroom is likely to be an effective method for preventing mycobacterial colonization.

Copper pipelines might be effective for preventing NTM colonization. Inkinen et al. (95) surveyed water samples supplied through a copper pipeline and a polyethylene pipeline. All the samples were collected from an office building in Rauma, Finland. The drinking water distribution network of the building connects to copper and cross-linked polyethylene in cold-water pipelines and to two hot-water pipelines (copper and polyethylene). After 1 year of operation, samples were collected at five different sites of the building, and a microbial community analysis using NGS was performed for water and biofilm samples. Surprisingly, *Mycobacterium* spp. sequences were absent from the copper pipeline samples, and they were detected only in the cold polyethylene pipeline water and biofilm samples. The identified *Mycobacterium* spp. operational taxonomic units were closely related to *M. terrae* and *M. nonchromogenicum* (95).

Chlorination is usually performed to disinfect drinking water treatment plants, and it is believed that the chlorination of drinking water could be effective to decrease the number of NTM pathogens. However, the ability of NTM to tolerate chlorine could be a great driving force that allows NTM to inhabit drinking water. In addition, there is an interesting report that showed that NTM were not found in water samples in which the concentration of residual chlorine was greater than 0.5 mg/L. At residual chlorine concentrations between 0.0 and 0.5 mg/L, NTM were found in 11.2% (*n* = 376) of the samples, including *M. gordonae* (*n* = 154, 41.0%), *M. fortuitum* (*n* = 144, 38.3%), and *M. peregrinum* (*n* = 26, 6.9%) (37). Many regional authorities require minimum chlorine concentrations at water treatment plants: Spain (1.0 mg/L), France (0.5 mg/L), Switzerland (0.1 mg/L), Italy (0.2 mg/L), and Morocco (0.2 mg/L) (CWWA n.d.). Japan requires a minimum of 0.1 mg/L at the tap, and actual values may range from 1.0 mg/L at the plant to 0.6–0.7 mg/L at the tap (96). The formation of mycobacterial biofilms has been reported inside the water main of a WDS (71). Therefore, further investigation is required to evaluate the effect and suitable concentration of chlorine in WDSs. In contrast, performing ozonation and filtration treatments at drinking water treatment plants (the ozone concentration multiplied by the contact time ranged from 4.2 to 13.3 min mg/L) does not reduce *M. avium* recovery (65).

Higher temperatures in household plumbing might effectively reduce NTM colonization. Households with water heater temperatures of <50°C were more likely to yield NTM (17/20, 85%) compared with households in which the water temperature was >55°C (6/15, 40%) (50). Heat susceptibility analyses at 50, 55, 60, and 70°C showed that *M. avium, M. chelonae*, and *M. xenopi* were more thermo-resistant than *L. pneumophila* (97). The authors determined the decimal reduction time (*D* value), which is the time needed to inactivate 90% of the bacterial population, at different temperatures. The *D* value of *M. avium* at 70°C was 2.3 s, and at 60°C, it was 240 s.

The prevention of NTM colonization and the elimination of these pathogens from infection sources are critical issues that must be solved rapidly. This requires the accumulation of reliable data to ascertain the effectiveness of dryness, copper pipeline usage, appropriate chlorine concentrations in drinking water, high temperature, and new elimination methods.

## Conclusion

Water, soil, and dust have been reported to be MAC niches. Environmental MAC tends to gather in households, and in these niches, household tap water, bathrooms, potting soil, and garden soil are infection sources. Mycobacterial transmission routes are believed to proceed from natural reservoirs to households (i.e., *via* WDSs). The global spread of pulmonary MAC disease might be caused by human activities, as individuals carry MAC organisms that concomitantly attach to their belongings and the environments they live in. Thus, global human mobility and trade may increase the global transmission of MAC *via* fomites. Epidemiological surveys that include WGS techniques should verify this hypothesis in the near future.

Our living environment is more comfortable and much cleaner than that of several decades ago. Social needs also seem to aim to create cleaner and safer environments. Medical treatments have improved greatly, which has increased longevity. In addition, compromised hosts can live normally outside of hospitals. While these social changes should be beneficial, comfortable environments may also hospitable to MAC because of the reduction of competitors by disinfection. To stop the increase of MAC prevalence and to prevent pulmonary MAC disease, our goal should be to create a comfortable environment for humans and an uncomfortable environment for MAC, as well as to develop new and effective treatments for MAC diseases. To achieve our goal, investigations of the ecology of the MAC in environment and its sources and routes of infection, as well as the development of new and effective elimination methods, including new disinfectants and new medical treatments, are urgently needed.

## Author Contributions

YN and TI drafted the manuscript, and YN and FM collected and reviewed the literature.

## Conflict of Interest Statement

The authors declare there were no financial or commercial conflicts of interest associated with the report of the present study.
